# Dissociating STAT4 and STAT5 Signaling Inhibitory Functions of SOCS3: Effects on CD8 T Cell Responses

**DOI:** 10.4049/immunohorizons.1800075

**Published:** 2019-11-20

**Authors:** Ji Young Hwang, John E. Holland, Kristine B. Valenteros, Yanbo Sun, Young-Kwang Usherwood, Andreia F. Verissimo, Jason S. McLellan, Gevorg Grigoryan, Edward J. Usherwood

**Affiliations:** * Department of Microbiology and Immunology, Geisel School of Medicine at Dartmouth College, Lebanon, NH 03755;; † Department of Computer Science, Dartmouth College, Hanover, NH 03755;; ‡ Institute for Molecular Targeting, Geisel School of Medicine at Dartmouth College, Hanover, NH 03755;; § Department of Biochemistry and Cell Biology, Geisel School of Medicine at Dartmouth College, Hanover, NH 03755

## Abstract

Cytokines are critical for guiding the differentiation of T lymphocytes to perform specialized tasks in the immune response. Developing strategies to manipulate cytokine-signaling pathways holds promise to program T cell differentiation toward the most therapeutically useful direction. Suppressor of cytokine signaling (SOCS) proteins are attractive targets, as they effectively inhibit undesirable cytokine signaling. However, these proteins target multiple signaling pathways, some of which we may need to remain uninhibited. SOCS3 inhibits IL-12 signaling but also inhibits the IL-2–signaling pathway. In this study, we use computational protein design based on SOCS3 and JAK crystal structures to engineer a mutant SOCS3 with altered specificity. We generated a mutant SOCS3 designed to ablate interactions with JAK1 but maintain interactions with JAK2. We show that this mutant does indeed ablate JAK1 inhibition, although, unexpectedly, it still coimmunoprecipitates with JAK1 and does so to a greater extent than with JAK2. When expressed in CD8 T cells, mutant SOCS3 preserved inhibition of JAK2-dependent STAT4 phosphorylation following IL-12 treatment. However, inhibition of STAT phosphorylation was ablated following stimulation with JAK1-dependent cytokines IL-2, IFN-α, and IL-21. Wild-type SOCS3 inhibited CD8 T cell expansion in vivo and induced a memory precursor phenotype. In vivo T cell expansion was restored by expression of the mutant SOCS3, and this also reverted the phenotype toward effector T cell differentiation. These data show that SOCS proteins can be engineered to fine-tune their specificity, and this can exert important changes to T cell biology.

## INTRODUCTION

Cytokines are soluble factors that have effects on both immune and nonimmune cell types and are critical for the differentiation of immune cells. Cytokine signal transduction must be tightly regulated to avoid inappropriate signaling and integrate multiple signals received from different soluble factors simultaneously. Suppressor of cytokine signaling (SOCS) proteins perform important functions attenuating signaling by multiple cytokines through JAK/STAT pathways.

In T cells, signals from cytokines facilitate the differentiation of effector cells appropriate for the nature of the immunological challenge. This is true for CD4 T cell differentiation into diverse specialized cell fates that help the B cell response (T follicular helper cells) or provide protection from intracellular (Th1) or extracellular (Th2, Th17) pathogens. In CD8 T cells, there is less specialization by function, but cells receive differing signals to become either terminally differentiated effector cells or less-differentiated memory cells with the potential to persist in the host long-term (1). Developingways to manipulate cytokine signaling to more easily customize T cell function would be of great benefit for adoptive immunotherapy.

SOCS3 is a potent suppressor of JAK/STAT signaling, and it has also been reported to affect signaling through the RAS/ERK(2), FAK (3), and NF-κB (4) pathways. Its promoter is methylated, reducing SOCS3 expression in most head and neck cancers (5) in addition to lung cancers (6), prostate cancers (7), and ulcerative colitis–related colorectal cancers (8, 9). This loss of inhibitory function leads to excessive activation of signaling pathways normally regulated by SOCS3, such as STAT3 and FAK, promoting tumor growth. SOCS3 targets both JAK1 and JAK2 (10) and inhibits signaling through many proinflammatory cytokines, such as IL-6, IL-1, and TNF-α (4, 11–13), which are produced in excess in inflammatory and autoimmune diseases. Expression of SOCS3, therefore, has therapeutic potential to reduce pathological signaling mediated by these cytokines. For example, adenovirus-mediated SOCS3 expression reduced the severity of joint pathology in rheumatoid arthritis models because of reduced responsiveness to inflammatory cytokines, in turn leading to reduced production of proinflammatory IL-6 and TNF-α and higher production of anti-inflammatory IL-10 (14, 15). Importantly, SOCS3 also targets cytokines necessary for CD8 T cell differentiation, including IL-12 (16) and IL-2 (2). Therefore, there is the potential to selectively tune cytokine signaling by engineering SOCS3 to repress signals inhibiting T cell function or terminal differentiation but preserve signaling leading to effector or memory differentiation.

Cytokine-signaling pathways affected by SOCS3 are determined mostly by SOCS3 binding to intracellular domains of certain cytokine receptor subunits (17, 18). This is because of the affinity for these receptor subunits being markedly greater than the affinity between SOCS3 and JAK proteins. SOCS3 is present in a complex with cytokine receptor/JAK proteins, and interactions with JAK proteins are centered around the GQM motif (10). Segments of three parts of SOCS3 are involved in SOCS3/JAK binding: the SH2 domain, the extended SH2 subdomain, and the kinase inhibitory region (19). The kinase inhibitory region of SOCS3 then acts as a noncompetitive inhibitor for JAK catalytic activity (10, 20, 21). Bound protein is also targeted for ubiquitination by the recruitment of elongins B and C and Cullin5 by the SH2 domain, leading to protein degradation (20, 22, 23). Based on crystallo-graphic structures of the SOCS3/JAK2 complex (19) and JAK1 (24), we modeled the SOCS3/JAK1 interaction to design a mutant SOCS3 with altered specificity. This mutant was designed to dissociate the inhibition of JAK1 and JAK2, preserving the JAK2 interaction while ablating JAK1 binding. We then tested the effects of expression of this mutant SOCS3 on the CD8 T cell response. Our work shows that it is possible to dissociate the inhibitory effects of this protein on STAT4 and STAT5 phosphorylation to abrogate its effect on IL-2 signaling but preserve inhibition of IL-12 signaling. This demonstrates the feasibility of changing the substrate specificity of SOCS proteins, which may be very useful in the design of novel cell-based immunotherapeutics.

## MATERIALS AND METHODS

### Modeling

To model the SOCS3-JAK1 complex, we aligned the JAK1 apo structure (24) (Protein Data Bank [PDB] identifier [ID] 5E1E) onto the SOCS3-JAK2 complex (19) (PDB ID 4GL9) and modified JAK1 segments mapping to the interfacial region (based on JAK2) to estimate changesthat occur to JAK1 upon binding. Specifically, the SOCS3-JAK1 complex was constructed by combining the SOCS3 structure fromthe SOCS3-JAK2 complex (chains G and I in 4GL9) with the JAK1 apo structure aligned to the interfacial region between SOCS3 and JAK2,defined as allresidues in JAK2 (chainC in 4GL9) whose Cα atoms are within 15 Å of any Cα atoms in SOCS3. Alignment was done using the “align” command in PyMOL (The PyMOL Molecular Graphics System, Version 1.8; Schrödinger, LLC). Because this alignment produces clashes in the interface between SOCS3 and JAK1, three segments of JAK1 abutting SOCS3 were remodeled based on the conformations of the equivalent JAK2 segments in the SOCS3-JAK2 complex: residues A1035–1045 (segment 1), A1051–1059 (segment 2), and A1086–1102 (segment 3) of JAK1, corresponding to residues C1008–1018, C1024–1032, and C1059–1076 of JAK2, respectively.

To remodel each interfacial segment of JAK1, the corresponding segment of JAK2 was first aligned to JAK1. Then, at both N- and C-terminal boundaries of each segment, the structure of JAK2 was “stitched” to the structure of JAK1, such that the resulting model transitioned from JAK1 into JAK2 and back into JAK1. The stitching was done by searching the PDB for short-chain fragments that could accommodate the transition (see below). Specifically, for the N terminus of segment 1, residue A1035 of JAK1 was stitched to residue C1011 of JAK2. For the C terminus of segment 1, residue A1042 of JAK1 was stitched to residue C1018 of JAK2. Similarly, for segment 2, residue A1051 of JAK1 was stitched to residue C1027 of JAK2 for the N terminus, and residue A1056 of JAK1 was stitched to residue C1029 of JAK2 for the C terminus. Finally, for segment 3, residue A1086 of JAK1 was stitched to residue C1062 of JAK2 for the N terminus, and residue A1099 of JAK1 was stitched to residue C1075 of JAK2 for the C terminus. Because JAK2 includes an insertion relative to JAK1 in segment 3 (after JAK1 residue A1093), additional modeling was needed. To generate backbone models for JAK1 around this site, we searched the PDB for a seven-residue fragment, the first two residues of which superimposed well onto residues A1092–1093 of modeled JAK1, and, simultaneously, the last two residues of which superimposed well onto residues A1097–1098 of modeled JAK1. This segment, originating from PDB code 2NYX residues A57–A63 (2NYX_A57–63), was then stitched into modeled JAK1, following the same procedure as in the above stitchings, by transitioning from JAK1 residues A1089–1092 to the middle three residues of the segment and back to JAK1 residues A1094–1097. Segment 2NYX_A57–63 was chosen based on having a low root-mean-square deviation (RMSD) to the overlapping JAK1 regions (0.743Å backbone RMSD over four residues) and having a proline at the middle position, corresponding to the proline at position A1094 of JAK1. The latter condition was enforced to account for proline’s unique backbone dihedral angle preferences.

To predict which mutations were most likely to stabilize the SOCS3-JAK2 complex and destabilize the SOCS3-JAK1 complex, 58 potentially relevant SOCS3 interfacial positions were selected manually and mutated to each possible amino acid except cysteine. These were positions 22–39, 41–43, 47–56, 59, 69–82, 92, 94–100, 102, 105, 120, 123, and 161 in chain G. Each mutation was modeled in both the SOCS3-JAK1 crystal structure and the SOSC3-JAK2 modelstructure. Ineachcase,allnearbyside-chainswererepacked, and the full structure was minimized using the *talaris2014* Rosetta scoring function in PyRosetta (25). The resulting energies were used to compute a specificity score as the energy of the minimized SOCS3-JAK1 complex minus the energy of the minimized SOCS3-JAK2 complex, with mutation T24P producing the highest specificity score. Mutations at position 24 were chosen for experimental characterization based on this observation. We also scored all mutations by our recently developed protein design method dTERMen (J. Zhou, A.E. Panaitiu, and G. Grigoryan, manuscript posted on bioRxiv), resulting in mutation T24D having the most advantageous shift in specificity toward JAK2.

### Stitching

Stitching entails searching the PDB for a fragment that can smoothly bridge a transition between two segments of structure. In all of the above examples, we were remodeling loops by replacing them with alternative conformations (deemed more representative of the modeled structure), which entailed two stitching events and either terminus of the loop being replaced. Suppose we have a structure P composed of two sections connected with a loop. Say P1 and P2 are the N- and C-terminal sections, respectively, and L is the intervening loop. We are interested in remodeling the geometry of this loop based on a different loop fragment F (taken from the PDB, as described above). The procedure starts off by initially superimposing F onto L in some reasonable way (above, this superposition came about by aligning JAK1 onto JAK2 via the interfacial region). The boundaries between the different segments are defined such that the four most C-terminal residues of P1 structurally overlap with the four most N-terminal residues of F and the four most C-terminal residues of F overlap with the four most N-terminal residues of P2. With this, we define several “anchor” residues (i.e., positions between which we will be seeking bridging fragments). Specifically, the fourth-most C-terminal residue of P1 is selected as the first anchor (a_1_), the fourth-most N-terminal residue of F is selected as the second anchor (a_2_), the fourth-most C-terminal residue of F is selected as the third anchor (a_3_), and the fourth-most N-terminal residue of P2 is selected as the fourth anchor (a_4_). In the above modeling description, the listed stitched residues refer to these anchors.

Using MASTER (26), the PDB is searched for two backbone fragments: one for transitioning between a_1_ and a_2_ (bb_12_) and another for transitioning between a_3_ and a_4_ (bb_34_). In particular, bb_12_ is identified as the four-residue fragment whose N- and C-terminal residues simultaneously superimpose (in terms of non-hydrogen backbone atoms) onto a_1_ and a_2_, respectively, with the lowest RMSD. Similarly, bb_34_ is chosen as the four-residue fragment whose N- and C-terminal residues optimally superimpose onto a_3_ and a_4_, respectively. The middle two residues of these fragments are not used in the search criterion [this is enabled in MASTER via “gapped” searches (26)], but because the segments have to be contiguous, this has the effect of requiring good stereochemistry when transitioning from one anchor residue to the other. In each case, if the RMSD between terminal residues of bb_12_ and a_1_a_2_ and the RMSD between the terminal residues of bb_34_ and a_3_a_4_ are both below a chosen cutoff (we used 0.1 Å in this study), the stitch is accepted, and a structure of S with bb_12_ swapped for the a_1_–a_2_ region and bb_34_ swapped for the a_3_–a_4_ is returned. If not, then F is repositioned by optimally aligning its N- and C- terminal residues simultaneously onto the C-terminal residue of bb_12_ and the N-terminal residue of bb_34_, respectively, and the above search procedure is repeated, returning new bb_12_ and bb_34_. The process is stopped when a stitch is accepted, which in all cases in this study occurred within several iterations.

### Cloning of murine SOCS3 gene into pCigar-FH-IRES-clover retroviral vector and 293T cell transfection

A plasmid containing SOCS3 cDNA was purchased from Dharmacon (Lafayette, CO) to amplify the coding region of SOCS3 with primers that contain restriction enzyme sequences (EcoRI and BamHI). The sequences of the primers were 5′-atcGAATTCCGCCACCatggtcacccacagcaagttt-3′ and 5′-agtacGGATCCttaaagtggagcatcatactgat-3′. The PCR products and pCigar vector (kind gift from Dr. Y. Huang, Geisel School of Medicine at Dartmouth) were digested with EcoRI and BamHI and ligated to create pCigar-FH-IRES-clover-SOCS3 (Supplemental Fig. 1). Mutant versions of SOCS3 were constructed by site-directed mutagenesis and inserted into the same vector. To produce retroviral supernatant, 293T cells were transfected with pCigar-FH-IRES-clover-SOCS3 and pCL-Eco, a plasmid encoding viral packaging proteins. The 293T cells were plated on a 10-cm dish, and 15 μmg of each plasmid DNA mixed with a calcium phosphate solution was added onto the cells. At 12 h posttransfection, media was replaced with fresh media, and the cells were incubated another 36 h before viral supernatant was harvested. Harvested viral supernatant was filtered using 0.45-μm syringe filters and stored at −80°C until use for T cell transduction.

For immunoprecipitation and Western blot analysis of SOCS3-JAK protein complex, wild-type (WT) and mutant SOCS3 were subcloned in to pCigar vector with a C-terminal Strep-tag II using a pair of primers that contain restriction enzyme sequences and the Strep-tag II sequence. The sequences of the primers used were 5′-atcGAATTCCGCCACCatggtcacccacagcaagttt-3′ and 5′-agtacGGATCCttaCTTCTCGAACTGAGGGTGGCTCCAGGCGCTGCCaagtggagcatcatactgat-3′.

JAK1 and JAK2 cDNA were subcloned into an expression vector (pCMV6-AN-DDK) that has built-in DDK sequence in the N terminus (Origene Technologies, Rockville, MD). The cDNA inserts of JAKs were amplified from the plasmids containing the cDNAs from Origene Technologies, using primers with restriction enzyme sequences (AscI and MluI) for ligation. The sequences of the primers used for JAK1 were 5′-GCCGGCGCGCCcatgcagtatctaaatataaaag-3′ and 5′-GCTTAACGCGTttattttaaaagtgcttcaaatcc-3′. The sequences of the primers used for JAK2 were 5′-TCGCCGGCGCGCCcATGGGAATGGCCTGCCTTACAATG-3′ and 5′-GCTTAACGCGTTCACGCAGCTATACTGTCCCGGATTTG-3′.

To study the interaction of SOCS3 and JAK proteins, 293T cells were transfected with different combinations of the plasmids encoding SOCS3-WT–Strep and Strep-tagged SOCS3T24D, A50H mutant (SOCS3-DH)–Strep, DDK-JAK1, and DDK-JAK2. At 48 h posttransfection, cells were harvested, and pellets were snap frozen and stored at 280°C prior to lysate preparation for immunoprecipitation and SDS-PAGE analysis.

### PCR site–directed mutagenesis

Mutated SOCS3 gene inserts for cloning were generated by two rounds of PCR. For the first round, forward and reverse PCR primers were designed to introduce one to three mismatched bases to create a mutation in the center of the primers with 20–25 complementary sequences on either side. These primers were paired with additional primers containing restriction enzyme sites (EcoRI and BamHI) on either end to produce two PCR fragments, which were then gel purified, mixed, and subjected to short PCR cycles to generate full-length SOCS3 gene fragments with the desired mutations. During the second round of PCR, the mutant fragments from the first round of PCR worked as templates. The primer pair with the EcoRI and BamHI sites, respectively, was used to produce a large amount of the gene fragments for restriction digests for subsequent ligation with pCigar vector that had been digested with the same enzymes.

### Preparation of cell lysates

Cell pellets were thawed in ice and resuspended in lysis buffer (100 mM Tris-HCl, pH 8, 150 mM NaCl, 1 mM EDTA) containing protease inhibitors (Bimake). Cell suspensions were sonicated using amplitude 20% and two cycles of 7 s and centrifuged at 10,000 × *g* for 10 min at 4°C to remove cell debris. Protein concentration of cell lysates was determined using the Bio-Rad Protein Assay Kit (Bio-Rad).

### Copurification of DDK-JAK1 and DDK-JAK2 with WT or mutant SOCS3–Strep

DDK-JAK1 and DDK-JAK2 were copurified with WT SOCS3–Strep or SOCS3-DH-Strep using a Strep-Tactin-XT Superflow High Capacity (IBA Lifesciences) system following the manufacturer’s protocol. Briefly, 1.75 mg of total protein from each lysate (final volume 700 μl) was loaded in a small disposable column packed with ~100 μl of resin and equilibrated with wash buffer (100 mM Tris-HCl, pH 8, 150 mM NaCl, 1 mM EDTA). After extensive washing, SOCS3-Strep proteins and respective interacting partners were eluted with elution buffer (wash buffer containing 50 mM biotin). Elution fractions were pooled and precipitated overnight at −20°C with 7:2 methanol/acetone (v/v). Immunoprecipitation and Western blot analysis for SOCS3-JAK interactions were performed in the Institute for Molecular Targeting at The Geisel School of Medicine at Dartmouth College.

### SDS-PAGE analysis and Western blot

Precipitated protein samples were centrifuged at maximum speed for 25 min at 4°C. Protein pellets were dried at 60°C for 10 min and resuspended in 1× SDS-PAGE Laemmli sample buffer containing 2.5% 2-ME. Proteins were resolved in a 12% Laemmli gel and transferred to a low-fluorescence PVDF membrane (Bio-Rad). The presence of WT or mutant SOCS3–Strep, DDK-JAK1, and DDK-JAK2 proteins in the elution fractions was detected in the same PVDF membrane using mouse Strep-Tag-II mAb (Novagen) and rabbit FLAG tag Ab (Sigma-Aldrich) (1:2000 dilution) as primary Abs and IRDye 680RD goat anti-mouse IgG (LI-COR Biosciences) and IRDye 800CW goat anti-rabbit IgG (LI-COR Biosciences) (1:12,000 dilution) as secondary Abs. Image analysis was performed using the Image Studio Lite Quantification Software (LI-COR Biosciences).

### Mice and Listeria infection

C57BL/6NCrl (B6) mice were originally obtained from Charles River Laboratories. CD45.1-expressing C57BL/6-Tg(TcraTcrb) 1100Mjb/Crl (CD45.1^+^ OT-I) mice were generated by crossing B6.SJL-*Ptprc*^a^*Pepc*^b^/BoyCrl (B6-Ly5.1; CD45.1^+^ B6 congenics) and OT-I mice. Mice were maintained under specific pathogen-free conditions in the Dartmouth Center for Comparative Medicine and Research. The Animal Care and Use Committee of Dartmouth College approved all animal experiments. The ActA^−^ LM-OVA was a generous gift of Dr. J. T. Harty (University of Iowa, Iowa City, IA). Seven-week-old mice were infected with 1 × 10^6^ CFU of LM-ActA^−^OVA retro-orbitally in 100 μl of PBS.

### T cell purification, differentiation, viral transduction, and adoptive transfer

CD8^+^ T cells were purified from the spleens of naive CD45.1^+^ OT-I mice using EasySep Mouse CD8^+^ T Cell Isolation Kit (StemCell Technologies) according to the manufacturer’s instructions. Single T cell preparations were >95% pure as determined by flow cytometry. Effector T cells were generated by activating naive T cells for32 h with plate-bound anti-CD3 (10 μg/ml;145–2C11; Bio X Cell), soluble anti-CD28 (5 μg/ml; 37.51; Bio X Cell), and rIL-2 (25 U/ml; National Cancer Institute). Cells were transduced (8 × 10^6^ cells/ml) with the different viral supernatants with polybrene (8 μg/ml), then cultured in RPMI 1640 with 10% FBS in the presence of IL-2 (25 U/ml) for 48 h. Effector CD8^+^ T cells (5 × 10^4^/mouse) were injected retro-orbitally into LM-ActA^−^OVA–infected recipients, and the splenocytes were collected on day 7 and day 28 postinfection.

### Cell preparation and flow cytometry

Single-cell suspensions from spleen were prepared by passing through cell strainers and resuspended in Gey’s solution (150 mM NH_4_Cl,10 mM KHCO_3_, and 0.05% phenolred) for 5 minto lyse red cells. Cell suspensions were then filtered through a 70-μm nylon cell strainer (BD Biosciences), washed, and resuspended in PBS with 2% bovine growth serum and 10 μg/ml Fc Block (2.4G2; DartLab) on ice for 10 min before staining with the following fluorochrome-conjugated Abs: anti–CD8-BV650 (53–6.7), anti–CD45.1-BV421 (A20), anti–CD127-PE (A7R34), anti–KLRG1-PE-Cy7 (2F1), anti–p-STAT1–PE (A15158B), and anti–p-STAT3–AF647 (13A3–1), all from BioLegend. For p-STAT5 staining, transduced cells were reseeded in IL-2–free media for 4 h, then cultured in RPMI with 10% FBS and anti–CD8-biotin (53–6.7; eBioscience) in the presence or absence of rIL-2 (50 U/ml; National Cancer Institute) for 10 min. Cells were fixed in 2% paraformaldehyde (5 min) and fixation reagent (15 min) (FIX & PERM Cell Permeabilization Kit; Thermo Fisher Scientific) and made permeable with ice-cold methanol (15 min) and permeabilization medium (FIX & PERM Cell Permeabilization Kit; Thermo Fisher Scientific), then stained with GFP tag Ab (Thermo Fisher Scientific), streptavidin-PE, and anti–p-STAT5-allophycocyanin (SRBCZX; all from eBioscience). Cells were analyzed with an Accuri C6 (BD Biosciences) or CytoFLEX S (Beckman Coulter) Flow Cytometers.

### Cytokine treatment and p-STAT staining

Forty-eight hours after transduction, cells were first reseeded in IL-2–free media for 4 h, then cultured in RPMI medium with 10% FBS and anti–CD8-biotin Ab (53–6.7; eBioscience) in the presence or absence of rIL-2 (50 U/ml) for 10 min to induce STAT5 phosphorylation. For STAT1 and STAT3 phosphorylation, cells were cultured in the presence or absence of IFN-α (5000 U/ml; 30 min; BioLegend) or IL-21 (200 ng/ml; 30 min; BioLegend), respectively, and anti–CD8-biotin was added for the last 10 min. Cells were then fixed in 2% paraformaldehyde (5 min) and fixation reagent (15 min) (FIX & PERM Cell Permeabilization Kit; Thermo Fisher Scientific) and made permeable with ice-cold methanol (15 min) and permeabilization medium (FIX & PERM Cell Permeabilization Kit; Thermo Fisher Scientific), then stained with anti-GFP Ab (Thermo Fisher Scientific), anti–p-STAT1-PE (A15158B), anti–p-STAT3-AF647 (13A3–1; all from BioLegend), anti–p-STAT5 allophycocyanin (SRBCZX), streptavidin-PE (all from eBioscience), and streptavidin-allophycocyanin (Invitrogen). Cells were analyzed with a CytoFLEX S (Beckman Coulter) Flow Cytometer.

### Western blot assay for phosphoSTAT4

Transduced (clover^+^) cells were sorted with a FACSAria cell sorter (BD Biosciences, Dartlab, Dartmouth-Hitchcock Medical Center) and reseeded in fresh media (RPMI 1640 medium supplemented with 10% FBS) for 6 h, prior to culture in the presence or absence of rIL-12 (2.5 ng/ml; 4 h; BioLegend) or pervanadate (0.5 mM Na_3_VO_4_ and 1.5 mM H_2_O_2_, mixed 7 min prior to use). Then cells were washed in HBSS, resuspended in ice-cold RIPA buffer with protease and phosphatase inhibitors for 1 h at 4°C, and the lysates were mixed with sample buffer and boiled at 100°C for 5 min. When ready for Western blot assay, equal amounts of protein were loaded onto 10% polyacrylamide gels and probed with purified anti–β-actin (2F1–1) and anti-STAT4 (15A1B41), all from Bio-Legend, and anti–p-STAT4 (pY693; 38/p-Stat4; BD Biosciences). The blots were then incubated with HRP sheep anti-mouse (Jackson ImmunoResearch).

### Statistical analysis

Two-way ANOVA Sidak or Dunnett multiple comparison test was used (GraphPad Prism Version 7.0). The *p* values < 0.05 were considered statistically significant.

## RESULTS

**FIGURE 1.**
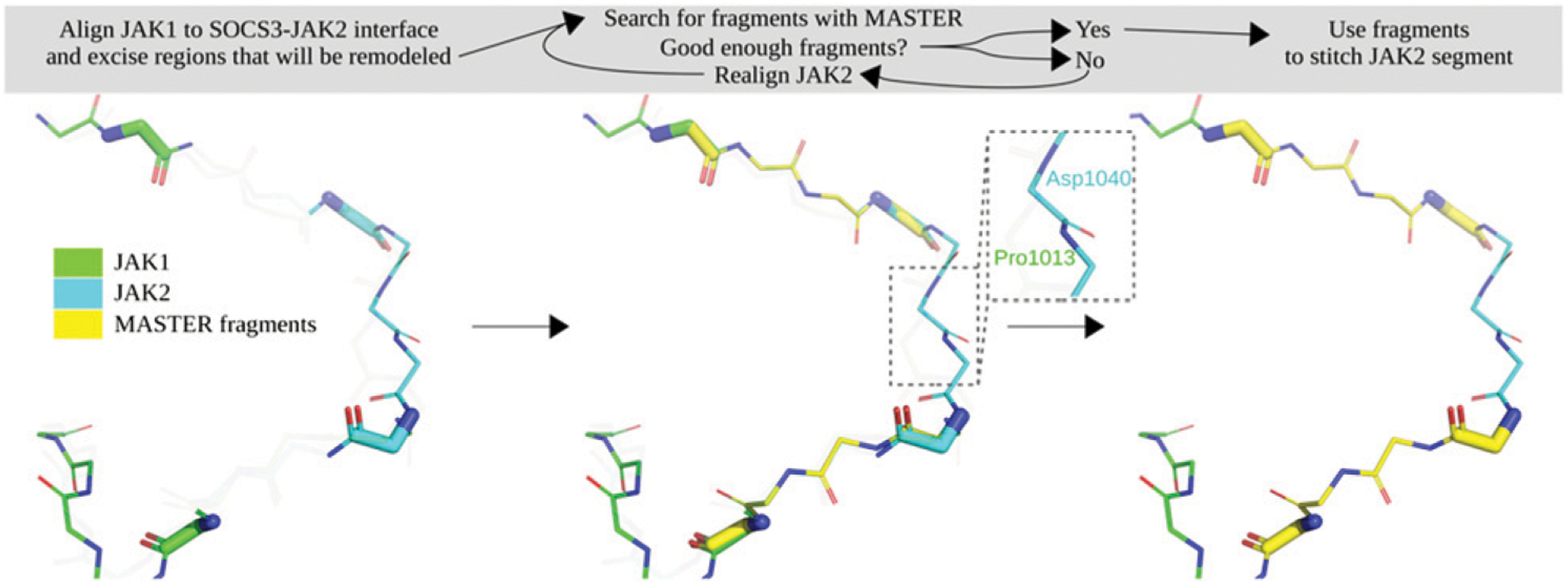
Summary of steps in the modeling of the SOCS3-JAK1 complex onto the published SOCS3-JAK2 complex. The boxed region shows Asp1040 and Pro1013, residues on JAK1 and JAK2 that interact with SOCS3, also identified in the diagram in Fig. 2.

**FIGURE 2.**
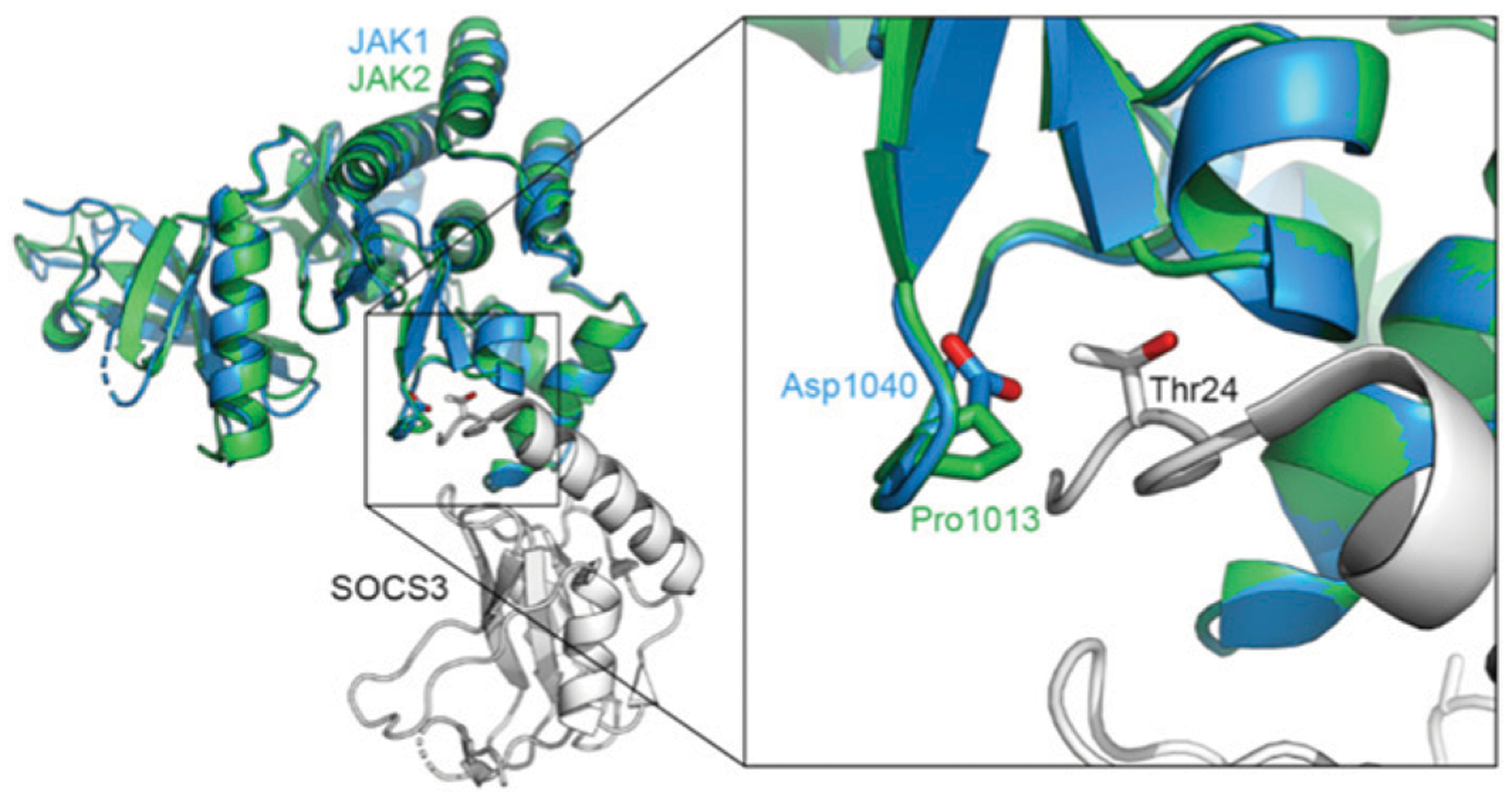
Model of the T24D mutation in SOCS3 complexed with JAK1 (blue) and JAK2 (green). Ribbon representation of the SOCS3-JAK1 complex model and the SOCS3-JAK2 complex crystal structure (PDB ID: 4GL9). The side-chain of residue Thr24 in SOCS3 is shown as sticks, as are the side-chains of Asp1040 and Pro1013 in JAK1 and JAK2, respectively. The T24D substitution is predicted to repel the negatively charged Asp1040 residue in JAK1.

**FIGURE 3.**
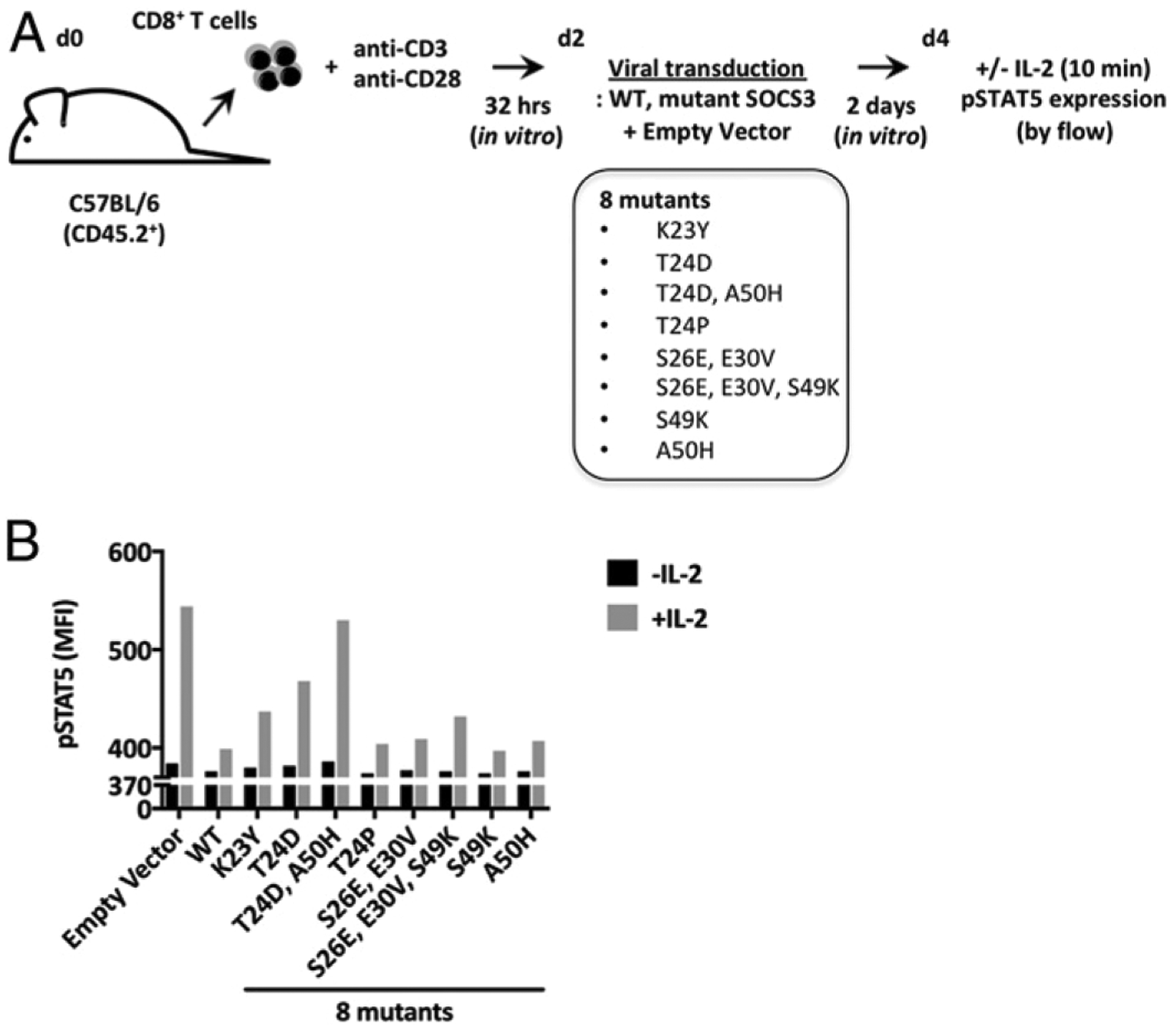
Screen of SOCS3 mutants to determine capacity to inhibit STAT5 phosphorylation. (**A**) Schematic showing experimental design and SOCS3 mutants tested; (**B**) CD8 T cells transduced with the mutants shown were incubated with IL-2 or no cytokine, then stained for intracellular p-STAT5. Mean fluorescent intensity is shown, gating on transduced (clover^+^) cells. Data are representative of two to three experiments.

The goal of this study was to design a SOCS3 mutant that retained the ability to inhibit IL-12 signaling through JAK2 but eliminated IL-2–signaling inhibition through JAK1 and to test if this affected effector or memory CD8 T cell differentiation. Although crystal structure is available for the JAK1/SOCS3 complex, we modeled this interaction using the published JAK2/SOCS3 complex (19) and the structure of JAK1 (24). We designed amino acid substitutions at the JAK1/SOCS3 interface designed to disfavor JAK1 interaction but not impede interaction with JAK2. The design process is described in detail in the *Materials and Methods* and summarized in Figs. 1 and 2, which show the key T24D mutation. Substitutions focused on two regions: one encompassing residues 23, 24, 26, and 30 and the second including residues 49 and 50. Some mutants included double or triple mutations, either in the same region or from both of these regions (Fig. 3A).

### Screening SOCS3 mutants for abrogation of IL-2–signaling inhibition activity

A key design feature of mutant SOCS3 was to disrupt inhibitory interactions with JAK1 and thereby allow unimpeded IL-2 signaling through phosphorylation of STAT5. We, therefore, screened the panel of SOCS3 mutants to identify those that had lost the ability to inhibit IL-2–induced STAT5 phosphorylation in CD8 T cells expressing SOCS3 mutants. To enforce expression over endogenous SOCS3 levels, constructs were designed expressing either WT or mutant SOCS3 in CD8 T cells using the pCigar-clover retroviral vector. Intracellular staining using a phospho-specific STAT5 Ab was used to detect p-STAT5 in clover^+^ cells after IL-2 treatment and measured using flow cytometry. Experimental design is summarized in Fig. 3A. After IL-2 treatment, there was an increase in p-STAT5 in cells transduced with the empty vector, but this was markedly reduced in cells transduced with WT SOCS3 (Fig. 3B, Supplemental Fig. 2). Whereas most mutants retained the ability to inhibit STAT5 phosphorylation, this was lost in the T24D mutant and, to a lesser extent and less reproducibly, in the K23Y mutant. Whereas the A50H mutant had no effect individually, the T24D, A50H double mutant consistently displayed higher STAT5 phosphorylation compared with the single T24D mutant. Therefore, the T24D, A50H double mutant was selected for further characterization and is referred to as “SOCS3-DH” hereafter.

**FIGURE 4.**
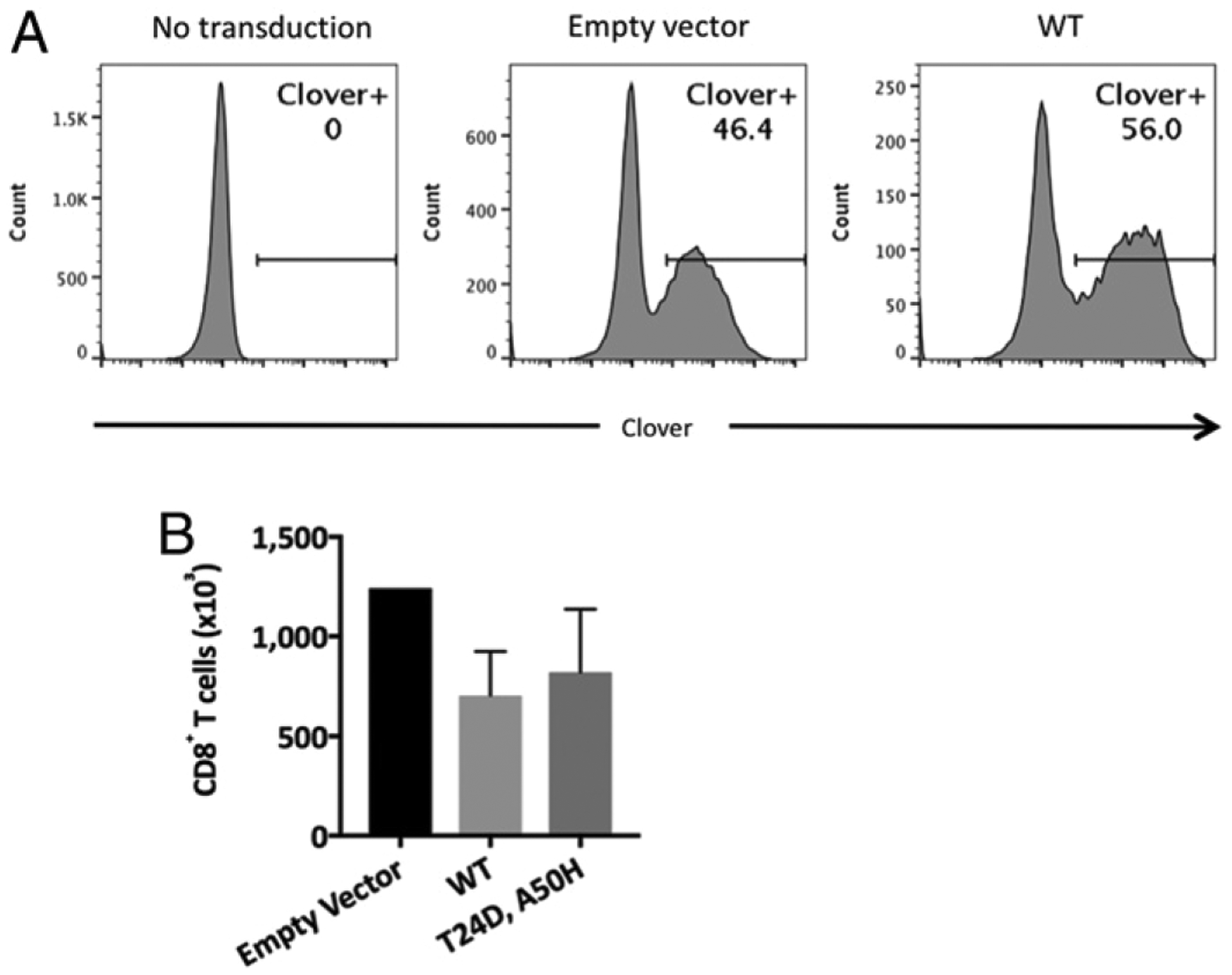
SOCS3 overexpression in CD8 T cells leads to comparable expansion in vitro. Purified CD8 T cells from OT-I mice were activated in vitro with anti-CD3 and anti-CD28 Abs, then transduced with retroviral vectors encoding clover plus WT SOCS3, SOCS3-DH, or an empty retroviral vector. (**A**) clover expression was measured after 48 h to test the efficiency of retroviral transduction. Numbers represent the percentage of events in the gate indicated. (**B**) CD8 T cell recovery from cultures 48 h after transduction. Representative data from two to three experiments.

Transduction efficiency was comparable with retroviral vectors containing either WT SOCS3 or the SOCS3-DH (Fig. 4A). At this time (48 h posttransduction, 80 h postactivation), we also observed comparable accumulation of CD8 T cells transduced with either the WT or mutant SOCS3 (Fig. 4B).

### SOCS3-DH has paradoxically enhanced binding to JAK1 compared with WT SOCS3

**FIGURE 5.**
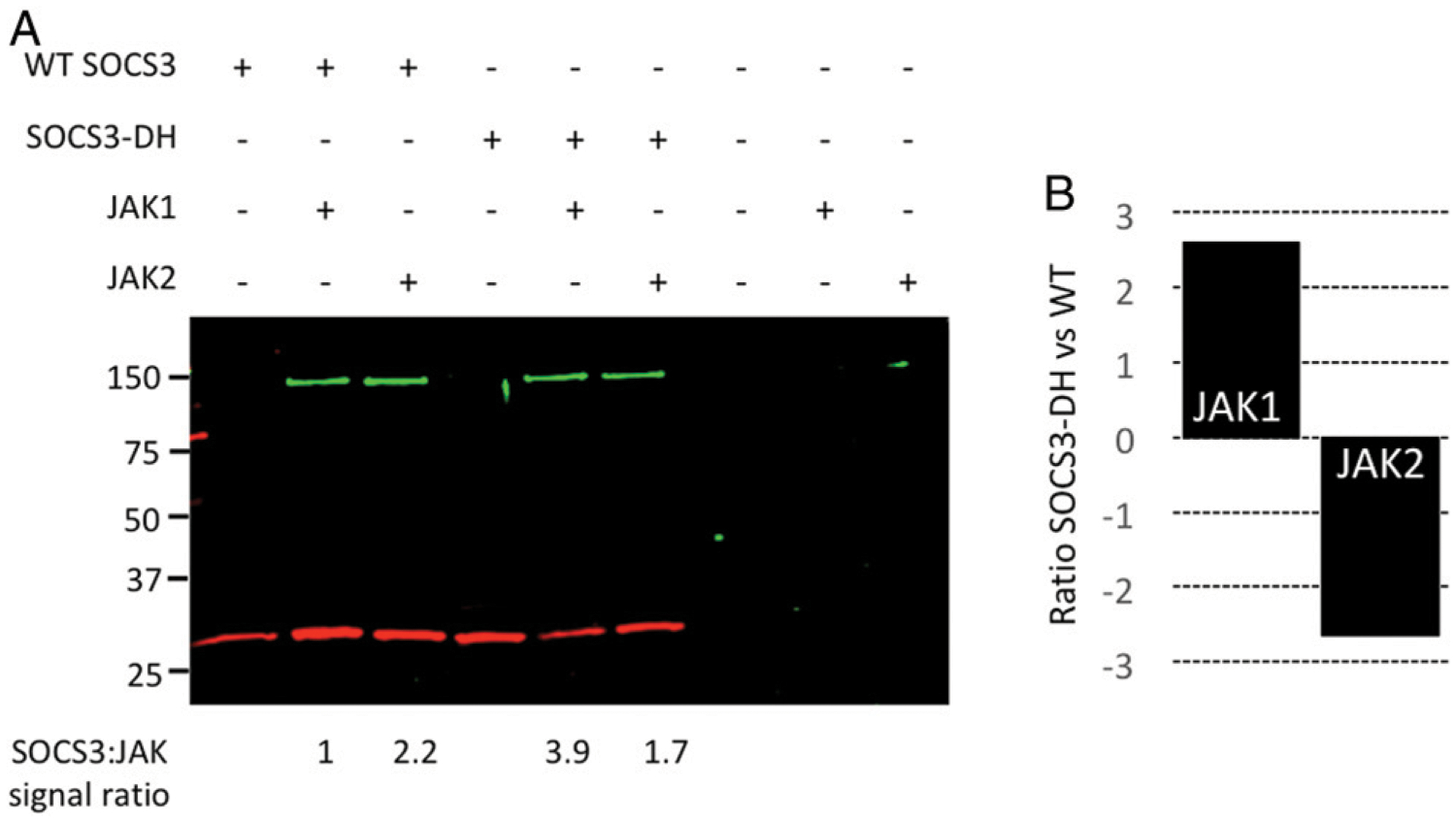
Copurification of JAK1 and JAK2 with WT SOCS3 and SOCS3-DH. (**A**) Immunoblotting analysis of the elution fractions resulting from purification of cell lysates expressing different combinations of WT SOCS3-Strep or SOCS3-DH–Strep and DDK-JAK1 and DDK-JAK2 proteins using Strep-Tactin-XT Superflow resin. JAK1 and JAK2 proteins (shown in green) were present in the elution fraction only in the presence of SOCS3 proteins (shown in red), indicating that their interaction is specific. Signal intensity for JAK1 and SOCS3 proteins present in the elution fraction was determined by image quantification using the Image Studio Lite Software. The ratio of JAK1 copurified with WT SOCS3 was defined as one and used to normalize the SOCS3/JAK signal ratios calculated for the remaining lanes. (**B**) Bar graph showing the ratio (SOCS3-DH/WT SOCS3) of the signal intensity for JAK1 binding and JAK2 binding. (A) Representative data. (B) Mean values from two experiments.

Having determined the optimal SOCS3 mutant that exhibited the desired loss of inhibition of STAT5 phosphorylation, we tested whether this was associated with the anticipated loss of JAK1 binding. JAK1 or JAK2 was transiently expressed in 293T cells, together with WTSOCS3 or SOCS3-DH, then SOCS3 was immuno-precipitated, followed by immunoblotting to detect the extent of binding to JAK1 or JAK2 (Fig. 5).

JAK1 and JAK2 proteins (Fig. 5A, shown in green) were precipitated only in samples containing SOCS3 proteins (shown in red), indicating that their interaction was specific. Signal intensity for JAKs and SOCS3 proteins was determined by image quantification. The ratio of JAK1 copurified with WT SOCS3 was defined as 1 and used to normalize the ratios calculated for the remaining lanes to assess the effect that mutations in SOCS3 protein had on binding to JAK1 and JAK2. The base of Fig. 5A shows the ratio of SOCS3 signal to JAK signal for the various groups. WT SOCS3 bound approximately twice as well to JAK2 compared with JAK1; however, SOCS3-DH bound better to JAK1 than to JAK2 (3.9 versus 1.7). Fig. 5B expresses these data in an alternate format, comparing the ratio of SOCS3-DH/WT SOCS3 binding to either JAK1 or JAK2. The unexpected finding was that, rather than abrogating JAK1 binding, the mutations introduced into SOCS3-DH enhanced binding to JAK1. As this mutant clearly abrogates inhibition of STAT5 signaling (Fig. 3), we concluded this increased binding, nevertheless, allows JAK1 phosphorylation of STAT5.

### Engineered SOCS3 retains inhibition of p-STAT4 after IL-12 stimulation

**FIGURE 6.**
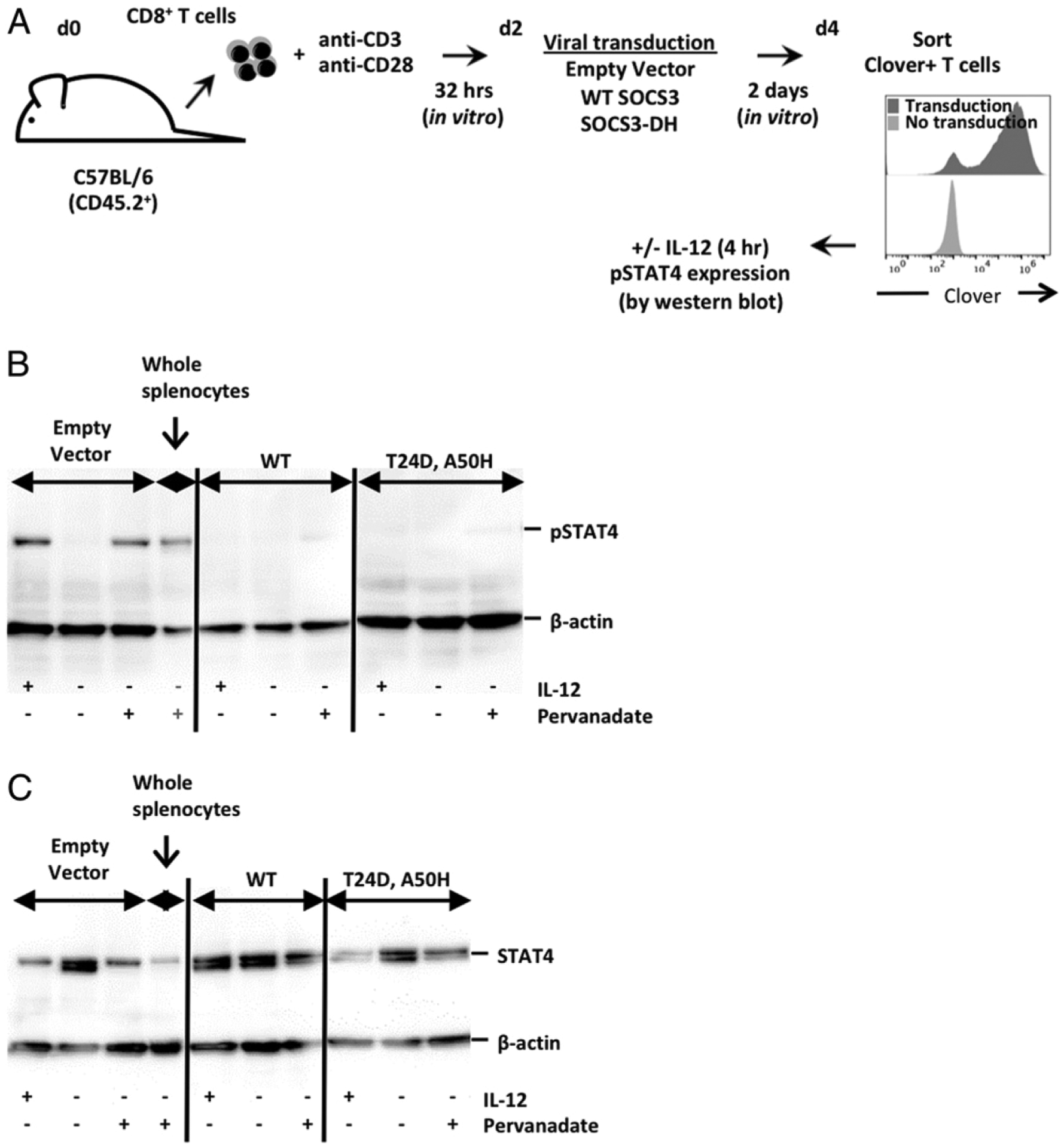
IL-12–induced p-STAT4 inhibition is intact in T24D, A50H mutant SOCS3–transduced cells. (**A**) Schematic showing experimental design; (**B** and **C**) T cells containing empty vector, WT SOCS3, or SOCS3-DH were sorted for CD8^+^clover^+^ cells, treated with IL-12 or no cytokine, then lysed and protein subjected to Western blotting to detect p-STAT4 (B) or total STAT4 (C). Whole spleen cells served as a positive control for IL-12–induced p-STAT4. Pervanadate is a protein tyrosine phosphatase inhibitor serving as chemical method to induce STAT4 phosphorylation. Representative data from two to three experiments.

An important part of our mutation strategy was to preserve the ability of SOCS3 to inhibit JAK2 and prevent signaling by IL-12 via STAT4 phosphorylation. We, therefore, transduced CD8 T cells with WT SOCS3 or SOCS3-DH and stimulated with IL-12 to measure the extent of STAT4 phosphorylation in clover^+^ cells (Fig. 6A). In these experiments, we used Western blot to detect both p-STAT4 and STAT4, as insufficient resolution was obtained using intracellular staining and flow cytometry. IL-12 stimulation resulted in p-STAT4 detection in cells transduced with the empty vector (Fig. 6B, lanes 1–2); however, this was abrogated in CD8 T cells expressing WT SOCS3 (Fig. 6B, lanes 5–6). Inhibition of STAT4 phosphorylation was retained in cells transduced with SOCS3-DH (Fig. 6B, lanes 8–9). Inhibition of STAT4 phosphorylation by both mutant and WT SOCS3 appeared highly efficient, as it was also suppressed after treatment with pervanadate, a phosphatase inhibitor that induces strong STAT phosphorylation, and used as a positive control (Fig. 6B, lanes 3, 7, and 10). Although expression of total STAT4 varied, it was detected in all experimental groups, confirming the absence of detection of p-STAT4 was not due to undetectable levels of the STAT4 protein (Fig. 6C). Therefore, mutations in SOCS3-DH that abrogate inhibition of IL-2 signaling through JAK1 do not adversely affect inhibition of IL-12 signaling through JAK2.

### Loss of inhibition of other JAK1-dependent cytokines by engineered SOCS3

**FIGURE 7.**
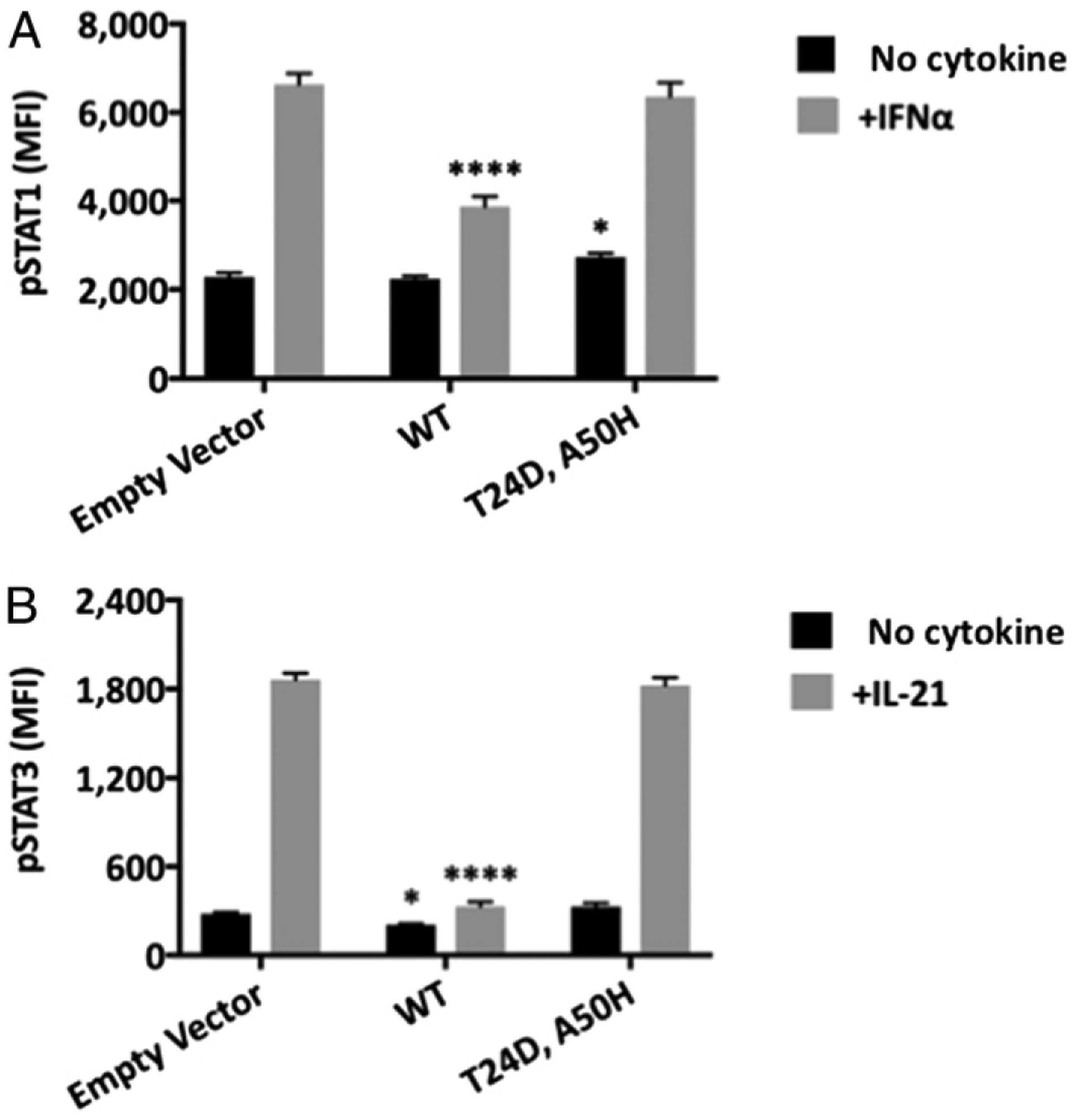
Mutant SOCS3 loses the ability to inhibit signaling by other JAK1-dependent cytokines. CD8 T cells were transduced with WT SOCS3, SOCS3-DH, or an empty retroviral vector, as described. Cells were either stimulated with (**A**) IFN-α, then stained for intracellular pSTAT1, or (**B**) IL-21, then stained for intracellular p-STAT3. Bars show mean fluorescent intensity (MFI) and SD. For statistical comparisons, no cytokine treatment groups were compared with each other across groups transduced with the three retroviruses, and cytokine-treated groups were similarly compared with each other. Representative data from two to three experiments are shown. *p < 0.05, ****p < 0.0001.

As mutant SOCS3 abrogated the inhibition of JAK1-dependent IL-2 signaling, we tested whether other JAK1-dependent cytokine responses were affected. We chose to test two other cytokines that have roles in T cell differentiation. IFN-3B1 signals through STAT1 and is important for providing “signal 3,” which is necessary for efficient memory CD8 T cell differentiation (27,28). IL-21 signals through STAT3 and has context-dependent functions in both effector and memory CD8 T cell differentiation(29). We, therefore, transduced CD8 T cells with WT SOCS3 or SOCS3-DH, then stimulated them with IFN-α or IL-21 and stained for either p-STAT1 (IFN-α) or p-STAT3 (IL-21). In both cases, cytokine addition increased p-STAT staining (Fig. 7A, 7B); however, this staining was significantly reduced when WT SOCS3 was expressed. SOCS3-DH exhibited p-STAT staining equivalent to empty vector–transduced cells, showing abrogation of suppression of cytokine signaling. This shows that other cytokine-signaling pathways relevant for CD8 T cell differentiation that signal through JAK1 also have inhibition by SOCS3 relieved by the mutant form of SOCS3.

### Effect on in vivo CD8 T cell responses of mutant SOCS3 expression

**FIGURE 8.**
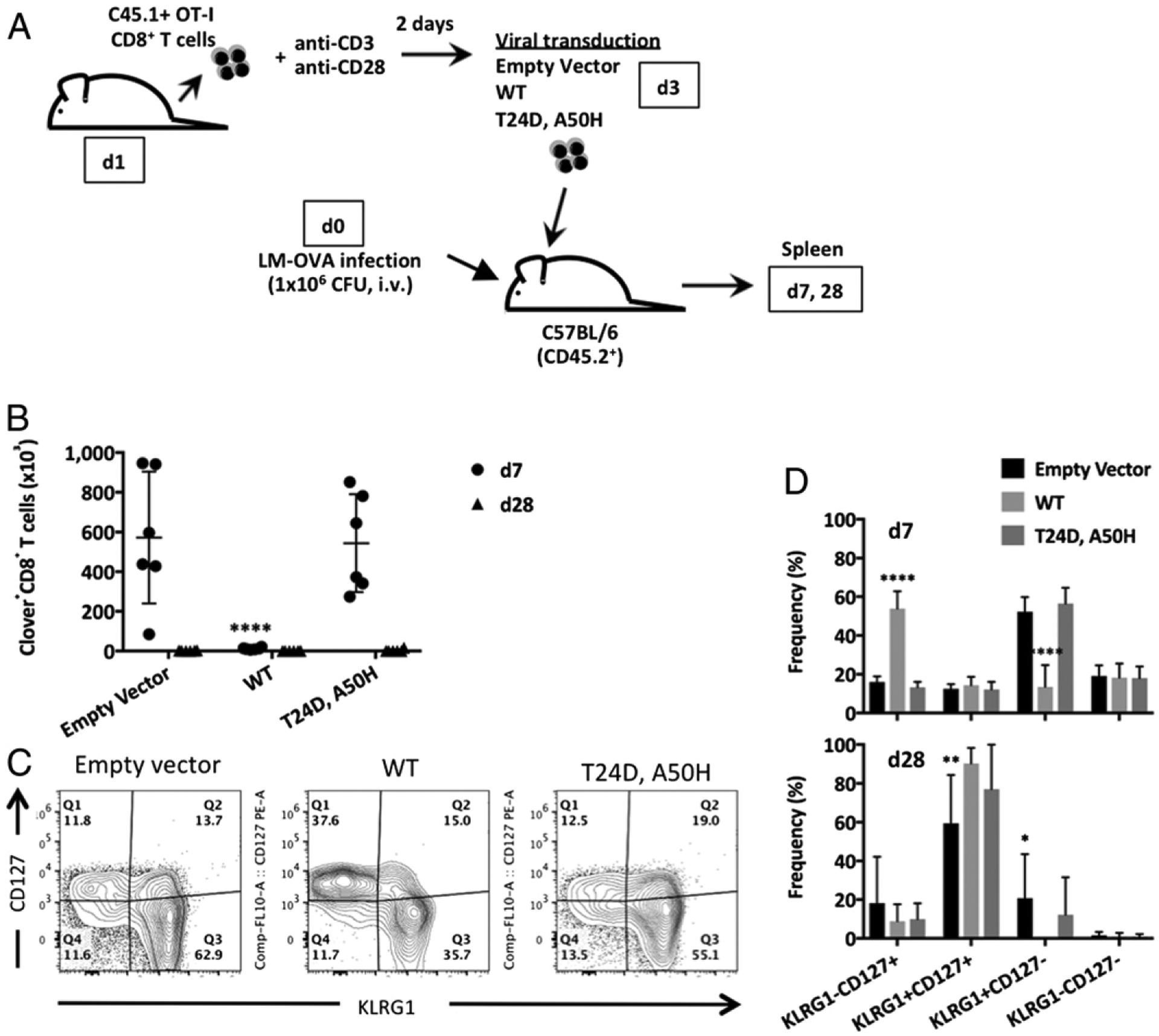
Fate of adoptively transferred cells following transduction with WT or mutant SOCS3. (**A**) Schematic of experimental design. Retrovirus-transduced OT-I cells were adoptively transferred into mice previously infected with LM-OVA. (**B**) Recovery of retrovirus-transduced OT-I cells from mice at day 7 or day 28 postinfection for groups adoptively transferred with cells transduced with empty vector, WT SOCS3, or SOCS3-DH. Each point represents data from an individual mouse. (**C**) KLRG1 and CD127 staining at day 7 to identify effector cells (KLRG1^hi^C127^lo^) and memory precursor cells (KLRG1^lo^CD127^hi^). Numbers show the percentage of events in each quadrant. (**D**) Bar chart showing KLRG1/CD127 phenotype of OT-I cells in experimental groups at day 7 (top) and day 28 (bottom) postinfection. Error bars show SD, bars show mean values. Data show representative data from two to three experiments. *p < 0.05, **p < 0.01, ****p < 0.0001.

Having shown modulation of cytokine signaling by SOCS3-DH, we tested the effect on CD8 T cell differentiation and function in vivo using an adoptive transfer infection model. OVA-specific OT-I–transgenic CD8 T cells were purified by negative selection with magnetic beads, activated with anti-CD3 and anti-CD28 Abs, then transduced with WT or mutant SOCS3. These cells were then adoptively transferred to congenic mice that had been infected 4 d before with *Listeria monocytogenes* encoding the OVA gene (LM-OVA, Fig. 8A). T cell survival and differentiation were then tracked at two different times after cell transfer (gating strategy shown in Supplemental Fig. 3). Day 7 posttransfer represents the effector response to LM-OVA, and day 28 postinfection allows measurement of the memory response. On day 7 postinfection, there were large numbers of empty vector–transduced OT-I cells in the spleen but a markedly lower number of cells transduced with WT SOCS3 (Fig. 8B). Cell numbers recovered from the SOCS3-DH–transduced group were equivalent to the empty vector group. These data are consistent with WT SOCS3 insulating CD8 T cells from IL-2 signaling necessary for sustained CD8 T cell expansion and abrogation of this inhibition with the engineered SOCS3. For both time points, we stained cells with Abs to KLRG1 and CD127 to detect terminally differentiated effector T cells (KLRG1^hi^CD127^lo^) or the precursors of memory cells (KLRG1^lo^CD127^hi^, Fig. 8C). At day 7 postinfection, a significantly greater proportion of memory precursor cells was detected in the WT SOCS3 population, and a significantly lower proportion of effector T cells was detected, when compared with the empty vector control (Fig. 8D). This effect was not observed in the SOCS3-DH group, which had a similar phenotype distribution to the empty vector group. By day 28 postinfection this bias toward the memory precursor phenotype was not evident, and in all groups, the dominant population was KLRG^hi^CD127^hi^ (Fig. 8D). At day 28 postinfection, as expected, memory populations were markedly smaller than at day 7 postinfection (Fig. 8B), and the enhanced memory precursor phenotype in the WT SOCS3 group did not translate into a larger memory population. These data indicate that SOCS3-DH relieves the repression of T cell expansion exhibited by SOCS3 transduction but does not affect the ultimate magnitude of the memory response.

## DISCUSSION

The first step in identifying mutations to SOCS3 that stabilize the JAK2/SOCS3 complex relative to the JAK1/SOCS3 complex was to computationally model the latter, for which no crystal structure was available. Thus, an accurate model of JAK1/SOCS3, particularly around the binding interface, was an essential prerequisite for effective protein design. It is difficult to judge, in the absence of direct structural validation, how good our model is in Fig. 1. Our data indicate that the mutations we designed do not inhibit SOCS3 binding to JAK1; indeed, binding is enhanced, but the mutations do prevent the inhibitory effect of JAK1 on STAT5 phosphorylation and IL-2 signaling. A possible reason for this is that the mutations we introduced disfavored the native bound state with JAK1; however, the three-dimensional conformation was altered in a way that resulted in a tighter binding to JAK1. We postulate this new binding conformation was then unable to inhibit JAK1 phosphorylation of STAT5 but retained the ability to inhibit JAK2 phosphorylation of target STATs.

Position 24 in SOCS3 marks the only region along the binding interface where JAK1 and JAK2 have different residues in contact with SOCS3. Unsurprisingly, therefore, Rosetta Design identified this position as the one at which mutations have the most consequence on the relative stability of JAK1/SOCS3 versus JAK2/SOCS3. The fact that mutation T24D consistently relieves the inhibition of pSTAT5 phosphorylation, whereas the WT and mutations at other positions do not, provides some support for our modeling of the interface. Although Rosetta identified mutation T24P as the one most likely to perturb specificity in the direction of JAK2/SOCS3, experimental characterization did not support such a shift for this mutation. In contrast, dTERMen, a protein design method recently developed in the Grigoryan laboratory(J. Zhou, A.E. Panaitiu, and G. Grigoryan, manuscript posted on bioRxiv), ranks T24D as the mutation at position 24 that would most significantly stabilize JAK2/SOCS3 relative to JAK1/SOCS3—a result that was borne out experimentally.

Mutations T24D and A50H appear to function synergistically in reducing pSTAT5 phosphorylation, in that A50H on its own has an undetectable effect while showing a significant effect in the context of T24D. Positions 24 and 50 are quite distant in structure, so this synergy does not appear to be a result of direct coupling between the residues. It may instead be that the T24D mutation sensitizes the structure, allowing additional minor changes to be observed. That is, it may shift the equilibrium toward JAK2 suf-ficiently such that further changes become functionally consequential when they may not be in the WT background.

The capacity of SOCS3 to inhibit JAK/STAT signaling makes it an attractive therapeutic target. Aberrantly strong JAK/STAT signaling in tumor cells can stimulate tumor growth, and SOCS3-mediated inhibition can reduce this effect and slow the growth of tumors. Approaches range from ectopic expression of SOCS3 in viral vectors (30) to the use of cell-permeable SOC3 protein (31). SOCS3 can also act as a negative regulator in immune cells, which, when suppressed, can stimulate immune responses that may be useful to counter tumors. However, given the effect of SOCS3 on multiple pathways, it may not always be desirable to release all these pathways from inhibition. For example, our data show SOCS3 inhibits signalingthrough IL-2, IL-12, IL-21, and IFN-α and likely also other cytokine signaling through JAK1 or JAK2, such as IFN-γ, IL-6, and IL-27. These cytokines stimulate T cell differentiation in differing ways, so blocking all by SOCS3 overexpression may not promote T cell differentiation as intended. In this study, we engineered SOCS3 such that it retains inhibition of cytokine signaling through JAK2 but lost the ability to inhibit cytokine signaling through JAK1. Whereas expression of WT SOCS3 suppressed responsiveness to IL-2, resulting in limited CD8 T cell expansion in vivo, cells expressing SOCS3-DH expanded normally and regained IL-2–signaling capability. This indicates that, although the interaction domain of SOCS3 with JAK1 is very similar to that with JAK2, at least in some cases, targeted mutations can allow the disruption of JAK1 inhibition without affecting inhibition of JAK2. Both with this protein and other members of the SOCS family, this may be a useful approach to surgically engineer these proteins to refine their specificity for optimal therapeutic use.

IL-12 signaling induces upregulation of the transcription factor T-bet, which is important for the effector functions of CD8 T cells, such as IFN-γ secretion. The IL-12–T-bet axis has been shown to induce terminal differentiation of CD8 T cells, favoring short-lived effector cells at the expense of memory precursor cells (32). Therefore, it was possible that the inhibition of IL-12 signaling by ectopic SOCS3 expression could favor generation of memory precursor cells. In our in vivo experiments, we observed skewing toward the memory precursor phenotype (CD127^hi^KLRG1^lo^) and away from terminally differentiated effector cells (CD127^lo^KLRG1^hi^), consistent with a prior report (33), albeit with a small population due to IL-2 unresponsiveness. Although the mutant SOCS3–transduced CD8 T cells expanded as well as control cells, they did not display the memory precursor phenotype. This difference is likely partly because of the fact that the mutations designed to abrogate inhibition of IL-2 signalingthrough JAK1 also allow signaling through other JAK1-dependent cytokines. Signaling by IL-21, IFN-α, and presumably other JAK1-dependent pathways is restored by the mutant SOCS3. Although it is not clear which of these pathways would prevent differentiation of memory precursor cells, type I IFN has been shown to promote effector cell differentiation (27, 28), and IL-21 can promote effector cell differentiation in a context-dependent manner (29, 34, 35). The absence of memory precursor skewing during suppression of IL-12 signaling was surprising, considering a previous study showing that IL-12–deficient mice infected with LM-OVA develop larger memory CD8 T cell populations (36). This report would indicate the absence of IL-12 signaling alone promotes memory differentiation at the expense of effector cells. However, in our system, in addition to the loss of IL-12 signaling, there is likely inhibition of other JAK2-dependent pathways, which could potentially counter the memory precursor– promoting activity. In addition, IL-2 signaling promotes glycolytic metabolism (37, 38), which is an important driver of effector differentiation, so abrogation of this signaling in the WT SOCS3 expressing cellsmay bea factorbiasingtowardtheobservedmemory precursor phenotype.

In conclusion, we engineered SOCS3, showing it is possible to functionally dissociate JAK1 inhibition from JAK2 inhibition. This released a major suppressive activity preventing IL-2 signaling while preserving SOCS3 inhibition of other signaling pathways. Our data from in vivo experiments show that inhibition of multiple cytokine-signalingpathways mediated by SOCS3 expression needs to be considered when designing novel immunotherapies using SOCS family proteins.

## Supplementary Material

1

## Abbreviations used in this article:

a_1_: first anchor
a_2_: second anchor
a_3_: third anchor
a_4_: fourth anchor
bb_12_: backbone fragment for transitioning between a_1_ and a_2_
bb_34_: backbone fragment for transitioning between a_3_ and a_4_
ID: identifier
PDB: Protein Data Bank
RMSD: root-mean-square deviation
SOCS: suppressor of cytokine signaling
WT: wild-type
