# Supplementary material for: Dissociating STAT4 and STAT5 Signaling Inhibitory Functions of SOCS3: Effects on CD8 T Cell Responses
Source: Immunohorizons. Author manuscript; Available in PMC 2020 Oct 20. (PMC7178138; doi:10.4049/immunohorizons.1800075)
Supplement: 1 [file NIHMS1583130-supplement-1.pdf]

Supplemental Figure S1

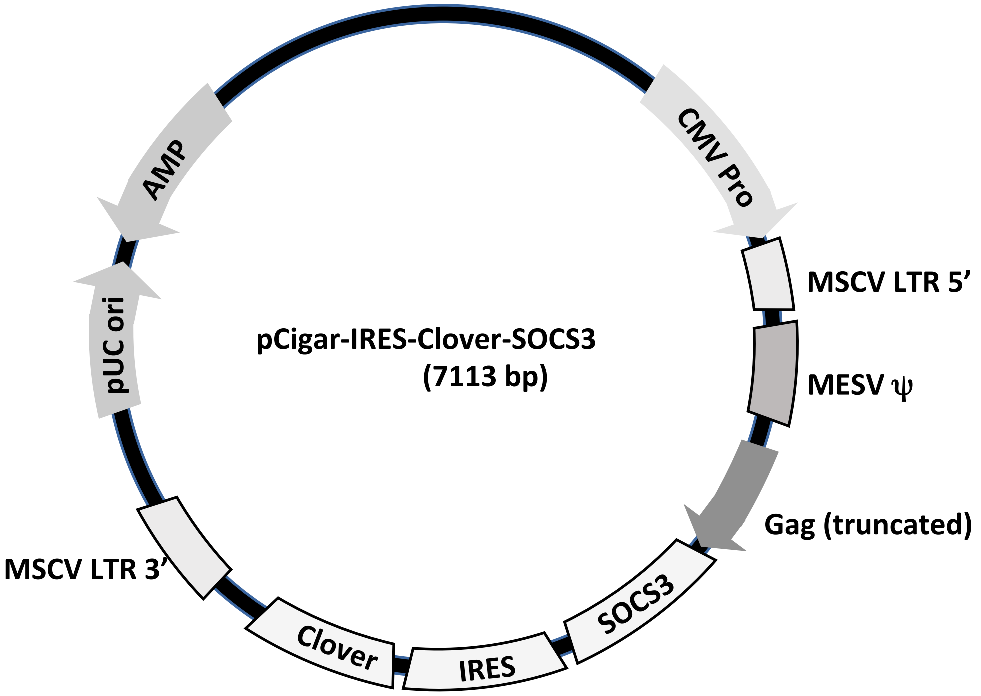

Figure S1. Map of the pCIGAR plasmid used to produce retroviruses containing WT or mutant SOCS3.

Supplemental Figure S2

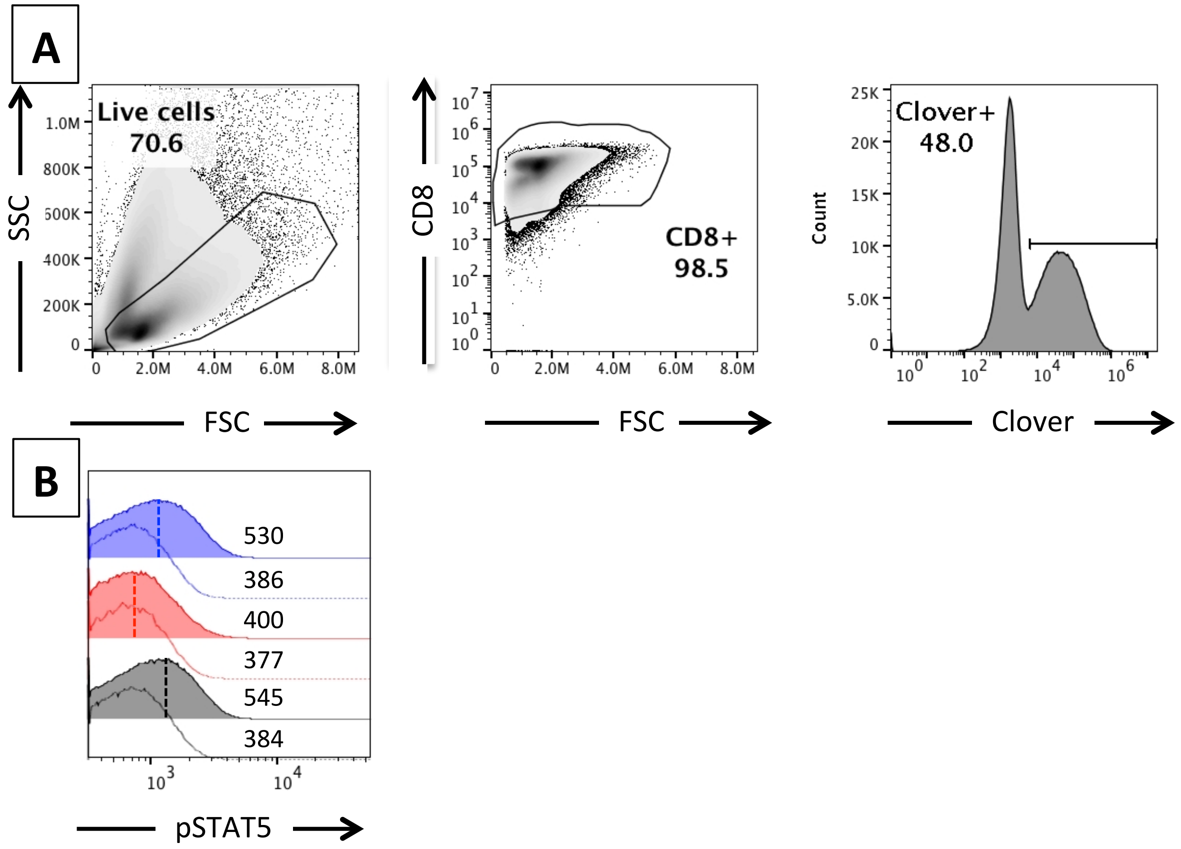

Figure S2. Intracellular staining for phosphoSTAT5 after IL-2 stimulation. (A) Gating strategy for identifying CD8 T cells successfully transduced with the relevant retrovirus; (B) histograms showing pSTAT5 levels with (filled histograms) and without (empty histograms) IL-2. Purple – T24D A50H SOCS3 mutant, red – WT SOCS3, grey – empty vector. Numbers show mean fluorescent intensity, marked by dotted vertical lines in filled histograms. Representative data from two experiments.

Supplemental Figure S3

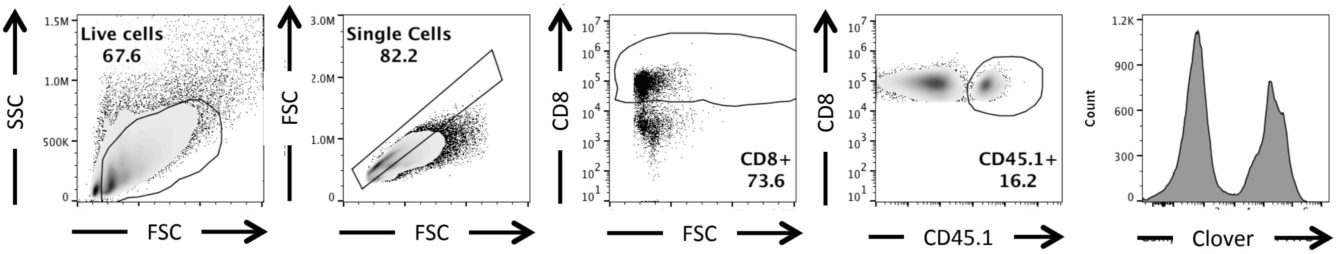

Figure S3. Gating strategy to identify adoptively transferred transduced OT-I cells . Cells were purified, transduced and adoptively transferred as described in Fig. 7A. Cells were then isolated from spleens at 7 or 28 days post-infection, and stained to identify $CD8^{+}CD45.1^{+}clover^{+}$ cells using the gating strategy shown. Representative data from two experiments is shown.
